# Sex differences in atrial fibrillation ablation in‐hospital outcomes from the National Inpatient Sample database 2016–2019

**DOI:** 10.1002/joa3.12831

**Published:** 2023-02-20

**Authors:** Biraj Shrestha, Julian Diaz Fraga, Bidhya Poudel, Anthony Donato

**Affiliations:** ^1^ Department of Medicine Tower Health System West Reading Pennsylvania USA; ^2^ Department of Medicine AMITA Health Saint Francis Hospital Evanston Illinois USA

**Keywords:** atrial fibrillation, catheter ablation, databases, risk, sex difference

## Abstract

**Background:**

Research has shown mixed results when comparing in‐hospital complications following atrial fibrillation ablation in women compared to men.

**Objectives:**

To better quantify sex differences and in‐hospital outcomes in atrial fibrillation ablation procedures and identify factors associated with poorer outcomes.

**Methods:**

We queried the NIS database from 2016 to 2019 for hospitalizations with a primary diagnosis of atrial fibrillation ablation and excluded patients with any other arrhythmias, ICD/pacemaker placement. We assessed demographics, in‐hospital mortality, and complications of women compared to men.

**Results:**

Admissions for atrial fibrillation were more common in females than males (849 050 vs. 815 665; *p* < .001). However, females were less likely to receive ablation (1.65% vs. 2.71%, OR: 0.60; 95% confidence interval: 0.57–0.64, *p* < .001), which persisted after adjusting for cardiomyopathy (adjusted OR: 0.61; 95% confidence interval: 0.58–0.65, *p* < .001). The primary outcome of in‐hospital mortality was not statistically different in univariate analysis (0.39% vs. 0.36%, OR: 1.09, 95% CI: 0.44–2.72, *p* = .84), finding that did not change when adjusted for comorbidities (adjusted OR: 0.94, 95% CI: 0.36–2.49). The complication rate in hospitalized patients following ablation was 8.08%. The total unadjusted complication rate was higher for females than males (9.58% vs. 7.09%, *p* = .001); however, it was not significant when adjusted for risks (adjusted OR: 1.23, 95% CI: 0.99–1.53, *p* = .06).

**Conclusion:**

Female sex is not associated with increased complications or death in a real‐world study of catheter ablation when results are adjusted for risks. However, females admitted with atrial fibrillation receive ablation less often than males during hospital admission.

## Abstract 2

Women make up a larger group of admitted atrial fibrillation patients than males. However, women with atrial fibrillation had a 39% lower likelihood of receiving catheter ablation in the hospital when compared to men. In‐hospital mortality was not statistically different between women and men in univariate and multivariate analyses. The total unadjusted complication rate was higher for women than men; however, it was insignificant when adjusted for risks. When controlled for risk factors, female sex was not associated with increased complications or in‐hospital death in a real‐world study.

## 1 INTRODUCTION

The incidence of atrial fibrillation has increased threefold over the last 50 years and is projected to reach 6 to 16 million by 2050 in the United States alone.^1^, ^2^, ^3^ Catheter ablation for atrial fibrillation has shown to significantly increase left ventricular ejection fraction (LVEF) and to lower mortality and heart failure hospitalization in patients with left ventricular ejection fraction (LVEF) ≤35% compared with medical therapy.^4^ Although atrial fibrillation is slightly less prevalent in women versus men (0.8% vs. 1.1%),^1^ women are significantly less represented in catheter ablation trials (13% to 37%).^4^, ^5^

Trials that address the safety and efficacy of catheter ablation in females compared to males have mixed results.^6^, ^7^, ^8^, ^9^, ^10^, ^11^, ^12^, ^13^, ^14^, ^15^, ^16^, ^17^, ^18^, ^19^, ^20^, ^21^ In most studies, females undergoing catheter ablation were older, with more comorbidities than males and higher CHADS_2_ ^7^, ^11^ and CHA_2_DS_2−_VASc scores,^6^, ^7^, ^11^, ^12^, ^13^, ^17^, ^18^, ^19^ possibly contributing to worsened outcomes.^7^, ^8^, ^9^, ^11^ Most importantly, studies were discrepant in the incidence of in‐hospital death, which was significantly higher in women than in men in some studies,^7^, ^8^ while other studies have shown no significant differences.^9^, ^10^, ^12^, ^13^, ^14^, ^19^, ^21^ Complication rates that were higher in females compared to males included pericardial effusion,^7^, ^8^, ^9^, ^10^ cardiac tamponade,^11^, ^16^ major bleeding,^11^, ^13^, ^21^ and cerebrovascular events,^7^, ^8^ while others have reported no difference in pericardial effusion,^12^, ^14^, ^15^, ^19^, ^20^, ^21^ cardiac tamponade,^12^, ^13^, ^15^, ^19^ major bleeding,^17^ and cerebrovascular events.^10^, ^12^, ^13^, ^15^, ^19^ It is unclear whether these complication rates contribute to overall in‐hospital death rates, length of stay, and costs.

To further evaluate this question, we queried a large US.inpatient database, the National Inpatient Sample (NIS), to analyze differences in demographics, comorbidities, and complications of atrial fibrillation ablation and their potential differences in incidence between women and men. Our research questions included: (A) How is the annual hospitalization utilization trend of atrial fibrillation ablation changing in women and men? (B) Are there differences in demographics and comorbidities between women and men undergoing catheter ablation that may be associated with in‐hospital mortality rates? (C) Are there any identifiable confounding variables (age and comorbidities) that could explain differences in outcomes in women compared to men?

## 2 METHODS

The NIS database was queried to conduct this retrospective study, reviewing records from 2016 to 2019. The database represents a 20% stratified sample of US hospital discharges and can be weighted to represent approximately 35 million discharges annually. The database is a part of the Health Cost and Utilization Project (HCUP).^22^ Each NIS database entry contains a unique hospitalization demographic, including age, sex, race, insurance status, primary and secondary procedure, hospitalization outcome, total cost, and length of stay.^23^

Hospitalization with atrial fibrillation (AF) was identified using the International Classification of Disease (ICD‐10) diagnosis codes I48.0, I48.1, I48.11, I48.19, I48.2, I 48.20, I48.21, and I48.91 in the primary diagnosis position. We excluded patients with a secondary diagnosis of atrial flutter, Wolf‐Parkinson‐White syndrome, atrioventricular nodal tachycardia, paroxysmal supraventricular tachycardia, paroxysmal ventricular tachycardia, sick sinus syndrome, and long QT syndrome based on the identification of their ICD and procedural codes (Table S1). Furthermore, we excluded hospitalized patients undergoing atrioventricular node ablation with pacemaker/ICD implantation during that admission. Records for hospitalized patients undergoing open ablation were also excluded based on its ICD procedural code (Table S1).^24^ We chose this strategy to identify only atrial fibrillation patients undergoing percutaneous catheter ablation in isolation. This methodology has been previously described in similar studies involving atrial fibrillation catheter ablation utilizing the NIS database.^9^, ^10^ We identified records of an ablation procedure using procedural codes 02563ZZ, 02573ZZ, 02583ZZ, 025S3ZZ, and 025T3ZZ. We excluded records for patients aged <18 years and those with missing sex, age, or in‐hospital mortality status. The study population was then divided into two comparative cohorts, women and men, based on NIS data elements.^23^

We calculated the annual utilization rate of atrial fibrillation catheter ablation procedures in the overall hospitalized population and in our comparative cohorts (in males and females) from 2016 to 2019 (Figure 2). We also collected demographic characteristics, including age, sex, race, and median household income according to the Zip code, primary payer (federal vs. private insurance), hospital‐level features such as hospital location (urban vs. rural), hospital bed size (small vs. medium vs. large), hospital region (Northeast, Midwest/North Central, South, and West), and hospital teaching status (non‐teaching vs. teaching). In addition, we collected comorbidities including common medical cardiovascular diagnoses, obesity, hypertension, diabetes, coronary artery disease, CKD stage, CABG history, history of coronary artery stent placement, hyperthyroidism, mitral valve stenosis, stroke, peripheral vascular disease, COPD, cancer history, autoimmune disease, cardiomyopathy (Table S2), and comorbidity burden (captured via Elixhauser Comorbidity Index). We also calculated CHADS_2_ and CHADS_2_‐VASc scores for each hospitalized patient based on the ICD codes (Table S3).

Our primary outcome was in‐hospital mortality. Our secondary outcomes were complications including pericardial effusion, cardiac arrest, pseudoaneurysm, arterial puncture, arterial embolism, hematoma, deep vein thrombosis (DVT), pulmonary embolism (PE), arteriovenous (AV) fistula, air embolism, diaphragmatic paralysis, phrenic nerve injury, post‐procedural shock, transient ischemic attack (TIA), post‐procedure stroke, and post‐procedural sepsis (Table S4). We did not analyze the data set for complications such as pulmonary vein stenosis that would have occurred long after the procedure and were unlikely to be related to the index procedure studied.

### 2.1 Statistical analysis

Among included patients, we created two mutually exclusive groups of females and males and analyzed characteristics of patient demographic and in‐hospital outcomes. Continuous variables were expressed as mean, and a 95% confidence interval was estimated. Categorical variables were represented in the percentage of the total population. We used weights provided by HCUP and svyset*; svy* functions in STATA to generate a national estimate of hospitalized US population from the observed hospitalization‐level data.^22^ Differences between groups were tested for continuous and categorical variables using t‐test and Chi‐square tests. *p*‐values of <.05 were considered statistically significant. We reported an unadjusted odds ratio for ablation rates between females and males and adjusted the odds ratio for cardiomyopathy in a bivariable logistic regression analysis. To estimate comorbidity burden, we used Elixhauser Comorbidity Index, which captures 38 comorbidity measures to calculate the risk of in‐hospital mortality and 30‐day all‐cause readmissions,^25^ which we computed using Statistical Analysis Software (SAS) version 9.4. We also conducted a Joinpoint regression analysis to observe the atrial fibrillation catheter ablation trend over included years using Joinpoint Trend Analysis Software version 4.9.0.1 (supplied publicly under Surveillance Research Program by National Cancer Institute).^26^

Univariate logistic analysis was done to assess the association of female sex with any outcomes, including overall complication rates, in‐hospital mortality, pericardial effusion, pericardial drain placement, postoperative shock, pseudoaneurysm, and post‐procedure stroke as compared to males. Subsequently, we performed multivariate logistic regression analysis for the same set of outcomes, adjusting for baseline factors significantly different in baseline characteristics by univariate analysis. We used the CHADS_2_ score and not the CHA_2_DS_2−_VASc score in our multivariable regression since CHA_2_DS_2−_VASc contains sex category as a component which would have affected the results of the logistic analysis. Results of regression analyses are expressed as odds ratios (ORs) with respective confidence intervals (CIs) and *p*‐values. The statistical software package STATA Version 14.2 was used to perform statistical calculations.

We did not seek an Institutional Review Board (IRB) approval as the data set contains no personally identifiable information.

## 3 RESULTS

**FIGURE 1:**
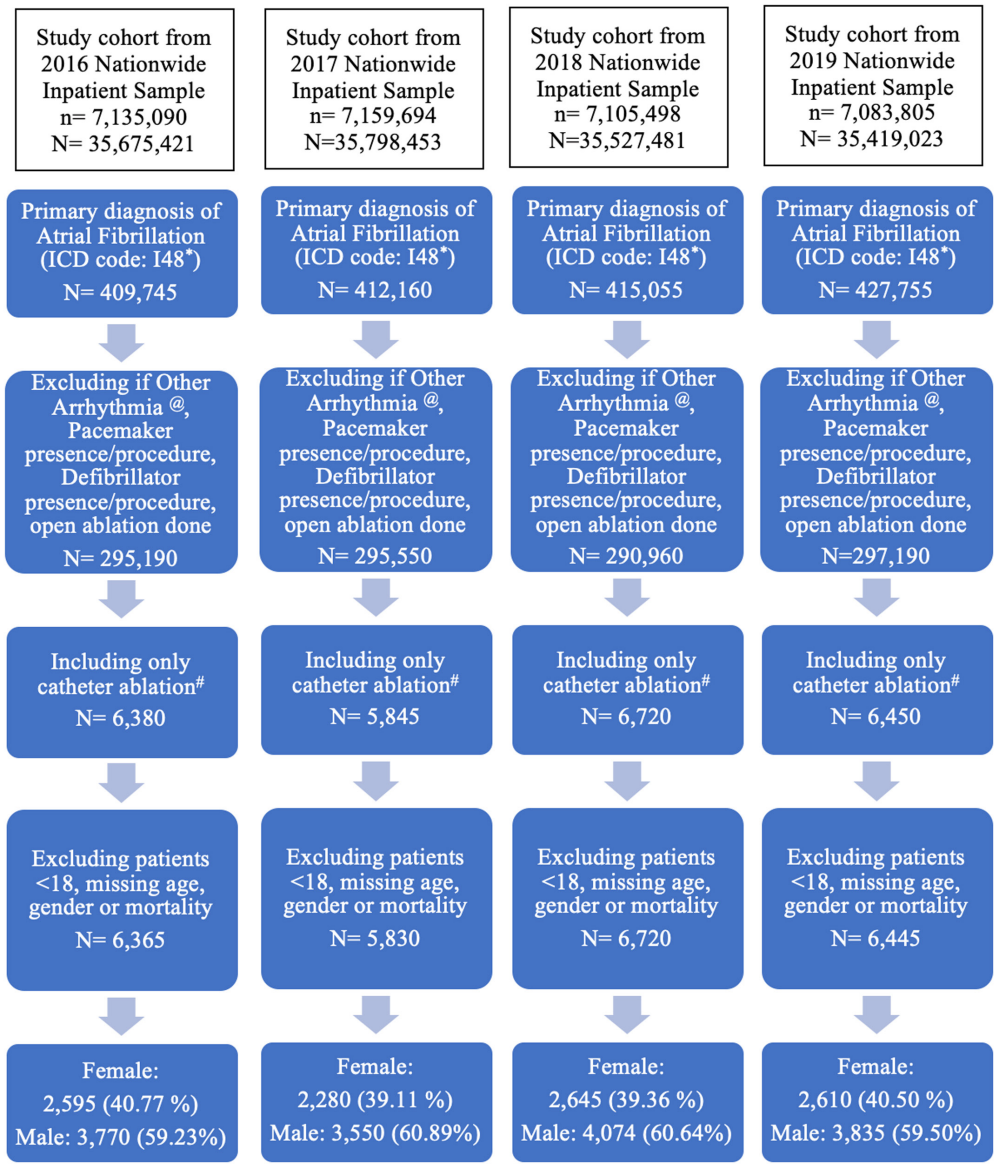
Diagram showing inclusion steps of patients using the Nationwide Inpatient Sample. ICD: International Classification of Diseases; *N*: Weighted sample data; *n*: Observed data. *See Table S1 for Atrial Fibrillation ICD‐10 code. ^@^See Table S1 for Other Arrhythmia ICD‐10 code. ^#^See Table S1 for Catheter Ablation ICD‐10 code.

**FIGURE 2:**
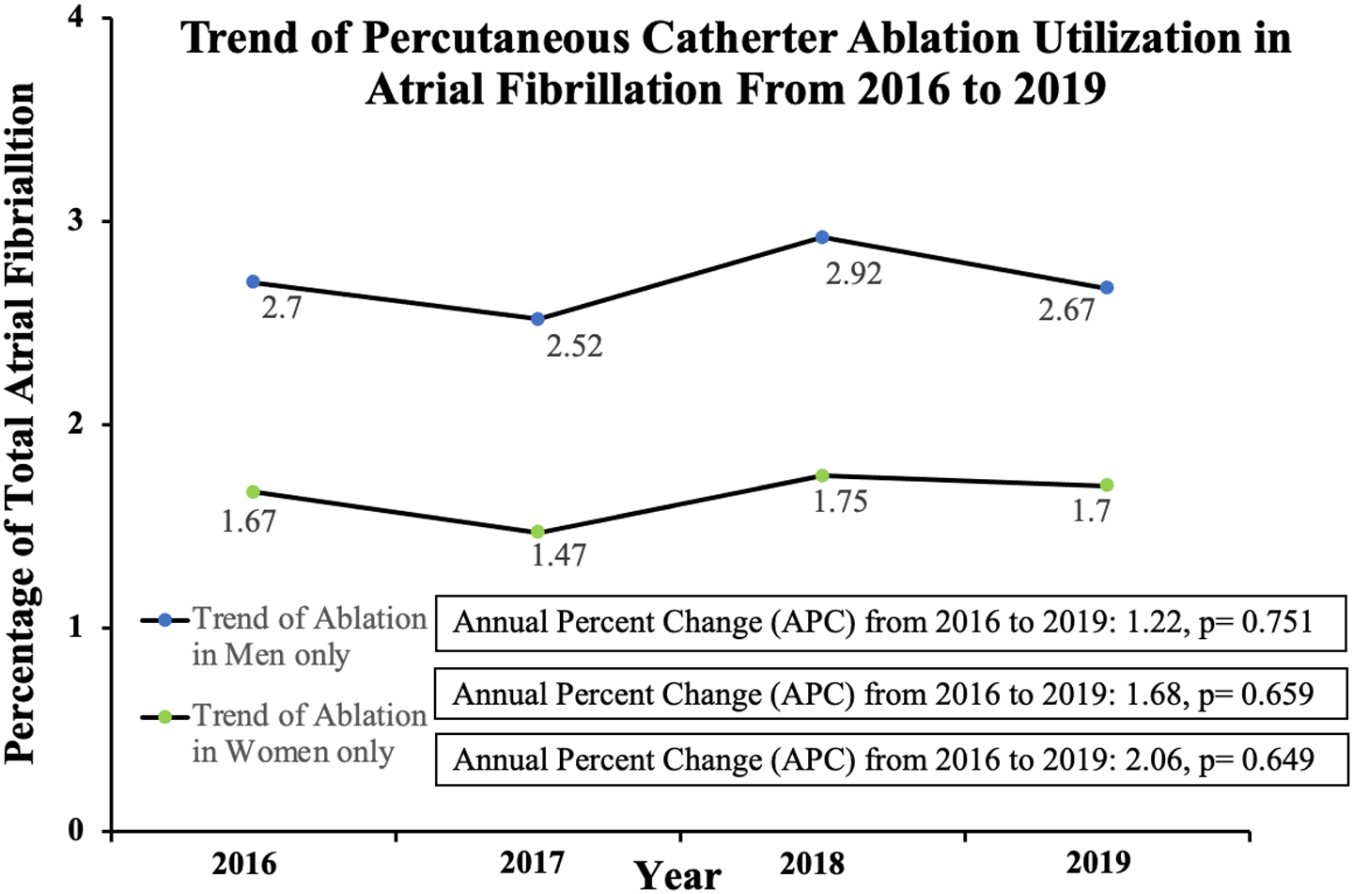
Diagram showing inclusion yearly trend of catheter ablation in males, female and overall hospitalized population from 2016 to 2019.

Out of the overall hospitalized patients weighted sample size of (*N*) = 142 420 378 hospitalizations (from the hospitalized patients sample size (*n*) = 28 484 087 observations), we identified 1 664 715 admissions for atrial fibrillation, which was more common in females (849 050 or 51% of total) than males (815 665 or 49% of total, *p* < .001). Atrial fibrillation ablation only was performed in 25 360 hospitalized patients based on our exclusion and inclusion criteria (Figure 1). Women with atrial fibrillation were less likely to undergo catheter ablation in the hospital when compared to men (1.65% vs. 2.71%, OR: 0.60, 95% confidence interval: 0.57–0.64, *p* < .001). This finding persisted after controlling for the higher prevalence of cardiomyopathy in males (adjusted OR: 0.61; 95% confidence interval: 0.58–0.65, *p* < .001). We found that the lower rates of atrial fibrillation ablation in females persisted even when stratified by age as seen in Table 3. We found that annual utilization of the ablation procedure did not significantly change through the years of our study (annual percent change of 1.68, *p* = .66) and did not change for either sex (Figure 2). Females undergoing catheter ablation were likely to be older (67.89 vs. 63.01 years, *p* < .001), were more likely to have BMI < 20 (0.49% vs. 0.0%, *p* < .001), a history of autoimmune disease (4.39% vs. 1.38%, *p* < .001), COPD (13.33% vs. 11.23%, *p* = .026), valvular heart disease (2.71% vs. 1.77%, *p* = .03), and prior stroke (9.48% vs. 6.60%, *p* < .001) as compared to males. Males had more coronary artery disease (CAD; 31.48% vs. 21.97%, *p* < .001) and more cardiomyopathy (17.04% vs. 13.98%, *p* < .001) as compared to female patients. Females had higher CHADS_2_ scores (1.68 vs. 1.48, *p* < .001) and CHA_2_DS_2−_VASc scores (3.64 vs. 2.30, *p* < .001) compared to males (Table 1).

**TABLE 1:** Baseline characteristics of the study population.

Baseline characteristics	Overall (%)	Male (%; *N* = 15 230)	Female (%; *N* = 10 130)	*p*‐value
	(*N* = 25 360)			
Atrial fibrillation ablation				
Mean age, (mean ± standard error; years)	64.96 (64.60–65.32)	63.01 (62.59–63.43)	67.89 (67.43–68.36)	<.001
Age (years)				<.001
18–49	8.00 (2030)	10.08 (1535)	4.89 (495)	
50–64	36.00 (9130)	42.65 (6495)	26.01 (2635)	
65–74	38.19 (9685)	34.96 (5325)	43.04 (4360)	
>=75	17.80 (4515)	12.31 (1875)	26.06 (2640)	
Gender				<.001
Male	60.06	100.00	0.00	
Female	39.94	0.00	100.00	
Race				.082
White	82.18 (20 840)	82.34 (12 540)	81.93 (8300)	
Black	4.51 (1145)	3.87 (590)	5.48 (555)	
Hispanic	5.82 (1475)	5.78 (880)	5.87 (595)	
Asian or Pacific Islander	1.62 (410)	1.61 (245)	1.63 (165)	
Native American	0.22 (55)	0.23 (35)	0.20 (20)	
Other	5.66 (1435)	6.17 (940)	4.89 (495)	
Comorbidity^a^				
Obesity	27.37 (6940)	26.53 (4040)	28.63 (2900)	.110
BMI < 20	0.20 (50)	0.00	0.49 (50)	<.001
Hypertension	73.52 (18 645)	73.60 (11 210)	73.40 (7435)	.871
Diabetes	23.82 (6040)	24.16 (3680)	23.30 (2360)	.475
Hypercholesterolemia	49.39 (12 525)	49.90 (7600)	48.62 (4925)	.357
Heart failure	25.51 (6470)	24.82 (3780)	26.55 (2690)	.162
Valvular disease	2.15 (545)	1.77 (275)	2.71 (270)	.031
Mitral valve stenosis	0.22 (55)	0.13 (20)	0.35 (35)	.109
CKD stage 3 or more	7.02 (1780)	7.06 (1075)	6.96 (705)	.890
Hyperthyroidism	0.30 (75)	0.26 (40)	0.35 (35)	.596
Peripheral vascular disease	6.11 (1550)	5.88 (895)	6.47 (655)	.388
COPD	12.07 (3060)	11.23 (1710)	13.33 (1350)	.026
Stroke	7.75 (1965)	6.60 (1005)	9.48 (960)	<.001
CAD	27.68 (7020)	31.48 (4795)	21.96 (2225)	<.001
CABG	4.20 (1065)	5.38 (820)	2.42 (245)	<.001
Stent	8.18 (2075)	10.28 (1565)	5.03 (510)	<.001
Cardiomyopathy	9.38 (3545)	17.04 (2595)	13.98 (950)	<.001
Cancer	1.46 (370)	1.71 (260)	1.09 (110)	.069
Autoimmune Disease	2.58 (655)	1.38 (210)	4.39 (445)	<.001
Disposition				<.001
Home	91.17	93.80	87.22	
Facility/others	8.46	5.84	12.39	
Died	0.37 (95)	0.36 (55)	0.39 (40)	
In‐hospital mortality (mean ± 95% Conf. interval; percentage)	0.37 (0.21–0.54)	0.36 (0.14–0.58)	0.39 (0.12–0.70)	.848
Length of stay (mean ± 95% Conf. interval; days)	2.79 (2.67–2.90)	2.61 (2.48–2.74)	3.04 (2.89–3.21)	<.001
Cost of care (mean ± 95% Conf. interval; USD)	31226.28 (30156.76–32295.79)	31555.69 (30323.17–32788.2)	30732.16 (29679.68–31784.65)	.086
CHA_2_DS_2_VASc (mean ± 95% Conf. interval)	2.83 (2.77–2.90)	2.30 (2.23–2.37)	3.64 (3.56–3.71)	<.001
CHADS_2_ (mean ± 95% Conf. interval)	1.56 (1.52–1.60)	1.48 (1.43–1.53)	1.68 (1.63–1.74)	<.001
Elixhauser comorbidity index for mortality (mean ± 95% Conf. interval)^b^	−2.21 (−2.36 to −2.06)	−1.79 (−1.96 to −1.63)	−2.84 (−3.08 to −2.59)	<.001

^a^ Comorbidities were coded using appropriate ICD‐10 In the secondary diagnosis field as per supplemental material online, Table S2.

^b^ *Elixhauser Comorbidity Software Refined for ICD‐10‐CM* 2021, Agency for Healthcare Research and Quality R, Rockville, MD (www.hcup‐us.ahrq.gov/toolssoftware/comorbidityicd10/comorbidityicd10.jsp).

**FIGURE 3:**
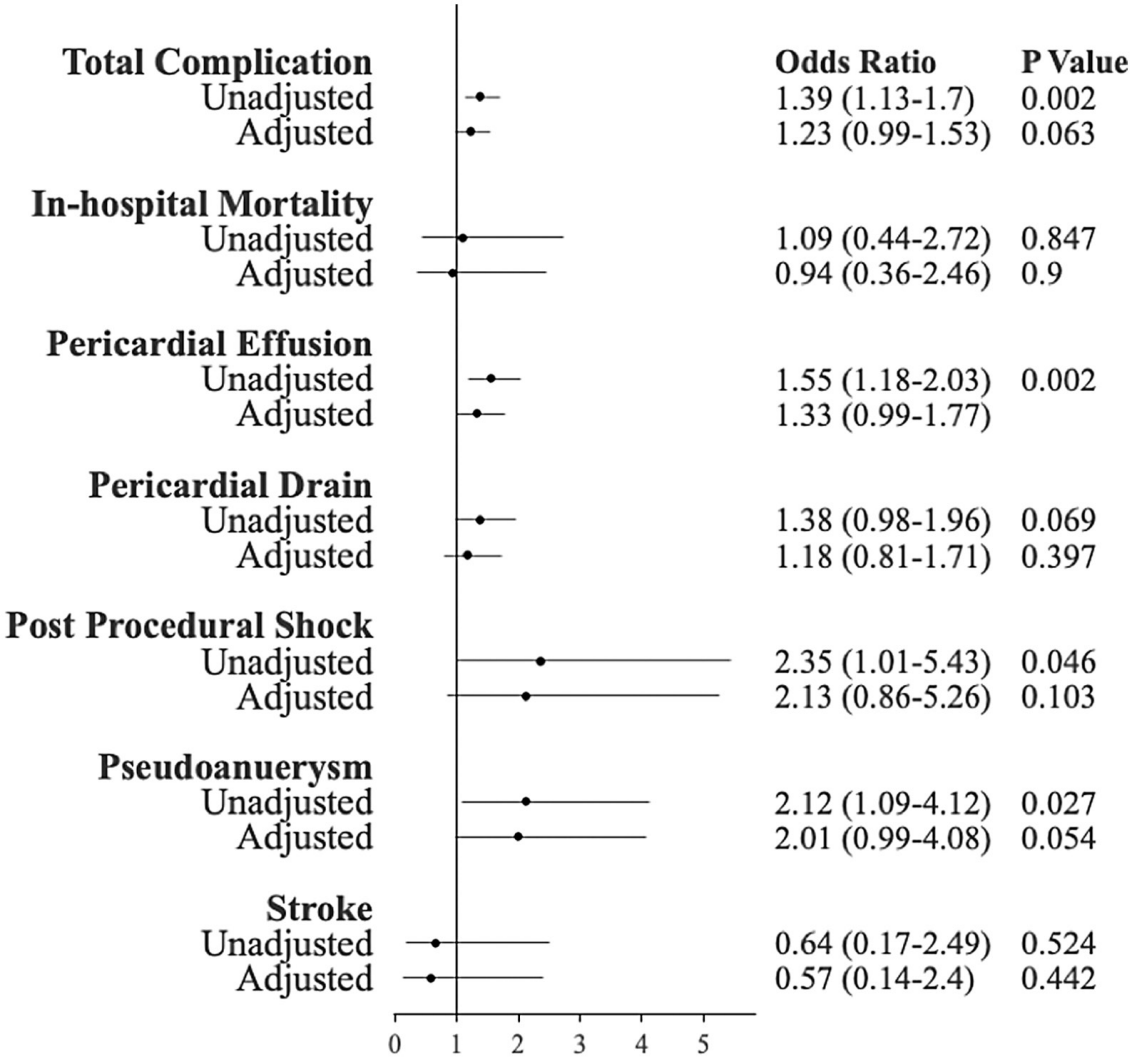
Unadjusted and adjusted odds ratios* for overall complication, in‐hospital mortality, pericardial effusion, pericardial drain, post‐operative shock, pseudoaneurysm, and post‐procedure stroke in females compared to males. *Adjusted for age, BMI < 20, history of valvular disease, stroke, coronary artery disease, cardiomyopathy, cancer, autoimmune disease, Elixhauser comorbidity index and CHADS_2_ score.

For the primary outcome, in‐hospital mortality for females compared to males was not statistically different in univariate analysis (0.39% vs. 0.36%, OR: 1.09, 95% CI: 0.44–2.72, *p* = .84), a finding that did not change when adjusted for comorbidities in multivariate analysis (adjusted OR: 0.94, 95% CI: 0.36–2.49). Length of stay was higher in females than in males (3.04 days vs. 2.61 days, *p* < .001), but no significant differences were seen in total hospital costs ($30 732 vs. $31 555 USD, *p* = .09). Females had a lower Elixhauser comorbidity index than males (−2.84 vs. −1.79, *p* < .001), which should predict a lower rate of hospital complications than males. However, we found that post‐procedural shock, pericardial effusion, and overall total complications were significantly greater in females compared to the male population undergoing cardiac ablation that resolved after adjustment for potential confounders (Table 2, Figure 3).

**TABLE 2:** Complications during hospitalization.

Complications^a^	Overall (%)	Male (%)	Female (%)	*p*‐value
	(*N* = 25 360)	(*N* = 15 230)	(*N* = 10 130)	
Cardiac complication				
Pericardial effusion	4.32 (1095)	3.58 (545)	5.43 (550)	.001
Pericardial drain	2.56 (650)	2.23 (340)	3.06 (310)	.058
Cardiac arrest	0.37 (95)	0.36 (55)	0.39 (40)	.848
Post procedure HF	0.10	0.13	0.05	.365
Vascular complication				
Pseudoaneurysm	0.71 (180)	0.49 (75)	1.04 (105)	.023
Arterial puncture	0.55 (140)	0.56 (85)	0.54 (55)	.941
Arterial embolism	0.32 (80)	0.30 (45)	0.35 (35)	.756
Hematoma	0.32 (80)	0.30 (45)	0.35 (35)	.755
Deep vein thrombosis	0.18 (45)	0.10 (15)	0.30 (30)	.101
Pulmonary embolism	0.16 (40)	0.13 (20)	0.20 (20)	.561
AV fistula	0.08 (20)	0.10 (15)	0.05 (5)	.541
Air embolism	0.02 (5)	0.00 (0)	0.05 (5)	.221
Respiratory complication				
Diaphragmatic paralysis	0.12 (30)	0.13 (20)	0.10 (10)	.740
Phrenic nerve injury	0.08 (20)	0.03 (5)	0.15 (15)	.152
Pneumothorax	N/A	N/A	N/A	
Hemothorax	N/A	N/A	N/A	
Circulatory complication				
Total post procedural shock	0.45 (115)	0.30 (45)	0.69 (70)	.040
Post procedural other shock	0.28 (70)	0.13 (20)	0.49 (50)	.016
Post procedural cardiogenic shock	0.20 (50)	0.16 (25)	0.25 (25)	.516
Major bleeding	1.28 (325)	1.25 (190)	1.33 (135)	.792
Bleeding requiring transfusion	0.26 (65)	0.26 (40)	0.25 (25)	.912
Neurological complication				
TIA	0.28 (70)	0.30 (45)	0.25 (25)	.746
Post procedure stroke	0.20 (50)	0.23 (35)	0.15 (15)	.521
Post‐procedural sepsis	0.28 (70)	0.33 (50)	0.20 (20)	.386
Anesthesia complication	N/A	N/A	N/A	
In‐hospital mortality	0.37 (95)	0.36 (55)	0.39 (40)	.848
Total complication	8.08 (2050)	7.09 (1080)	9.58 (970)	.001

^a^ Complications were coded using appropriate ICD‐10 In the secondary diagnosis field as per supplemental material online, Table S4.

**TABLE 3:** Differences in atrial fibrillation ablation between females and males.

Age	Female		*p*‐value	Male		*p*‐value
	No ablation	Yes ablation		No ablation	Yes ablation	
18–49	97.44 (18 830)	2.56 (495)	<.001	97.38 (56 990)	2.62 (1535)	<.001
50–64	97.28 (94 185)	2.72 (2635)	<.001	96.30 (169 185)	3.70 (6500)	<.001
65–74	97.36 (160 670)	2.64 (4360)	<.001	96.70 (156 245)	3.30 (5340)	<.001
≥75	99.21 (330 670)	0.79 (2640)	<.001	98.88 (165 495)	1.12 (1875)	<.001
Total	604 355	10 130		547 915	15 230	

## 4 DISCUSSION

This study identified that utilization of in‐hospital catheter ablation in females with atrial fibrillation was lower than in males (1.65% vs. 2.71%, adjusted OR: 0.61; 95% confidence interval: 0.58–0.65, *p* < .001), which were in line with previous study which showed that females received less inpatient catheter ablation.^27^, ^28^, ^29^, ^30^ Female patients in this study were older than males (average age 67.89 vs. 63.01 years, *p* < .001), similar to previous studies in which mean age for females ranged 60.9–67.9 years compared to 56 to 63.3 years for males.^7^, ^8^, ^9^, ^11^, ^12^, ^13^, ^14^, ^15^, ^17^, ^18^, ^19^, ^20^, ^21^ Other authors note both older age and lower referral rates for ablation procedures in females.^31^ Several factors postulated by others for late referral include patient factors (downplaying symptoms, personal/family situation) and gender‐specific higher individual refusal rates.^32^ Females in our study had higher CHA_2_DS_2−_VASc scores (even when accounting for additional points for female sex) but also significantly higher CHADS_2_ scores, similar to previous studies,^6^, ^7^, ^11^, ^12^, ^13^, ^17^, ^18^, ^19^ indicating that they potentially had higher stroke risk and could possibly benefit more from the restoration of sinus rhythm. We saw no significant difference in insurance status that could explain differences in procedures and noted more federal insurance in females than males in our study.

Complication rates in all hospitalized patients following ablation were 8.08%, similar to the ranges reported in other trials (5.67%–8.85%).^11^, ^13^, ^15^, ^18^ The total unadjusted complication rate was higher for females than males (9.58% vs. 7.09%, *p* = .001); however, it was not significant when adjusted for risks (adjusted OR: 1.23, 95% CI: 0.99–1.53, *p* = .06). This was despite a lower overall comorbidity burden in females as determined by the Elixhauser comorbidity index, contrary to previous studies.^7^, ^8^, ^9^, ^11^ Other authors had identified that females had higher complications than males attributed to higher comorbidities and later stage presentation.^11^, ^15^ Studies have shown that female patients are more symptomatic at presentation for atrial fibrillation than males.^13^ Moreover, anatomic differences, including smaller femoral size, vascular anatomy difference,^33^ and smaller left atrial diameter/geometry,^34^, ^35^ were thought to increase the risk of hematoma, puncture, and pericardial effusion. This data set did not find an association between female sex and increased complications when controlled for risk factors. These findings align with the recently published CABANA trial.^18^ Our observation was different from the previously observed study using a comparable NIS database, which can be partially explained by the fact that the ICD‐9 code for catheter ablation used by those authors was nonspecific as it was common for both atrial and ventricular ablation.^9^, ^10^ Most importantly, our study showed a similar in‐hospital mortality rate between men and women. Although two studies have shown higher mortality rates in women,^7^, ^8^ other studies have shown no significant differences.^9^, ^10^, ^12^, ^13^, ^14^, ^19^, ^21^ Our higher overall in‐hospital mortality rate for both sexes of 0.37% (compared to 0.03% to 0.32% reported in clinical trials)^11^, ^13^, ^14^, ^19^ may reflect the real‐world experience in actual patient populations that are not highly selected in clinical trials.

The strengths of our study are the size of the database used, national representation, and appropriateness of the database to study hospitalization outcomes. HCUP data is a large and validated database for which the differences observed are likely clinically relevant. We acknowledge some limitations, most of which are inherent to the nature of administrative databases and reliance on billing codes. We, however, have done the due diligence of using validated ICD codes to identify hospitalizations and comorbidities, adjusted our analyses for known modifiers of clinical associations. Despite these, unmeasured confounders could have influenced the results due to the non‐randomizable nature of retrospective data. Specifically, we could not control for the skill or experience of the catheter operator or hospital. In addition, because the NIS does not survey medications, we could not make adjustments for the use of anticoagulation, antiplatelet drugs, or antiarrhythmics that all may have influenced positive and negative outcomes. We also could not identify recurrence of atrial fibrillation as there is no specific ICD codes to denote this. We also could not identify the underlying cause of mortality. Finally, the unit of measurement of this database is a hospital occurrence, not an individual patient; we would be unable to control for one patient being responsible for more than one ablation admission occurrence. Post hoc power calculations using our death rate differences between women and men suggest we would need a sample size of 1 744 666 to completely rule out a type 2 error (with 90% power), suggesting that this study's sample size (n = 25 360) was not large enough to capture a small statistical difference in this population if present. However, this study was large enough to detect overall complications. Therefore, we believe the complication rates unique to the actual procedure could be relied upon to assess real‐world complication rates accurately.

## 5 CONCLUSION

Catheter ablation for atrial fibrillation is a safe and effective technique. Female sex is not associated with increased complications or death in real‐world experience when controlled for risk factors. However, there still seems to be a lower utilization rate than expected and a later age at presentation in females for ablation procedures. More study is needed regarding whether personal, physician or other factors contribute to later referral for females for catheter ablation.

## FUNDING INFORMATION

This project reports no source of funding.

## CONFLICT OF INTEREST STATEMENT

The remaining authors have nothing to disclose.

## ETHICS STATEMENT

As the database contains no patient identifiable information, we did not get patient consent or ethical approval.

## CLINICAL TRAIL REGISTRATION

Retrospective study from large nationally available database hence we did not get any registration.

## Supporting information


Table S1–S4
