# Supplementary material for: Sex differences in atrial fibrillation ablation in‐hospital outcomes from the National Inpatient Sample database 2016–2019
Source: J Arrhythm. 2023 Feb 20;39(2):149–58. doi: 10.1002/joa3.12831 (PMC10068935; doi:10.1002/joa3.12831)
Supplement: Supplementary file 1 — Table S1–S4 [file JOA3-39-149-s001.docx]

**Supplementary Table 1:** ICD 10 codes for Inclusion/Exclusion Criteria for Atrial Fibrillation ablation

| **Co-morbidities** | **ICD-10 Codes in Secondary field** |
| --- | --- |
| Atrial Fibrillation^@^ | "I480", "I481", "I4811", "I4819", "I482", "I4821", "I4891", "I4820" |
| Atrial Flutter^@^ | "I483", "I484", "I4892" |
| Paroxysmal tachycardia^@^ | "I47", "I470", "I471", "I472", "I479" |
| Premature Beat^@^ | "I491", "I492", "I493", "I494", "I4940", "I4949", "I498", "I499", "I495" |
| Excitable Syndrome^@^ | "I456", "I458", "I4581", "I4589" |
| Jervell-Lange-Nielsen syndrome^@^ | "I4581" |
| Pacemaker Presence^@^ | "Z950", "Z4501", "Z45010", "Z45018", "Z4502" |
| Pacemaker Procedure^@^ | "0JH604Z", "0JH605Z", "0JH606Z", "02H40JZ", "02H43JZ", "02H43NZ", "02H60NZ", "02H63JZ", "02H70JZ", "02HK0JZ", "02HK0NZ", "02HK3JZ", "02HK3NZ", "02HL0JZ", "02HL0NZ", "02HL3JZ", "02HL3NZ", "02HN4JZ", "02PA0NZ", "02PA3NZ", "02WA0NZ", "02WA3NZ", "02WA4NZ", "02WAXNZ", "02H40NZ", "02H60JZ", "02H63NZ", "02H70NZ", "02H73JZ", "02H73NZ", "02HN0JZ", "02HN3JZ", "0JH604Z", "0JH606Z", "0JH634Z", "0JH636Z", "0JH804Z", "0JH806Z", "0JH834Z", "0JH836Z", "02H44JZ", "02H44NZ", "02H64JZ", "02H64NZ", "02H74JZ", "02H74NZ", "02HK4JZ", "02HK4NZ", "02HL4JZ", "02HL4NZ", "02PA4NZ", "0JH605Z", "0JH607Z", "0JH635Z", "0JH637Z", "0JH805Z", "0JH807Z", "0JH835Z", "0JH837Z", "0JH606Z" |
| Defibrillator^@^ | "Z95810", "Z4502" |
| Defibrillator Procedure^@^ | "0JH607", "0JH607Z", "0JH608", "0JH608Z", "0JH609", "0JH609Z", "0JH60F","0JH60FZ", "0JH638", "0JH638Z", "0JH639", "0JH639Z", "02H40KZ", "02H43KZ","02H60KZ", "02H70KZ", "02HK3KZ", "02HL0KZ", "02HN0KZ", "02HN3KZ", "02HN4KZ", "02H63KZ", "02H73KZ", "02HK0KZ", "02HL3KZ" |
| Open Ablation procedure^@^ | "02560ZZ", "02570ZZ", "02B60ZZ", "02B70ZZ", "02T80ZZ", "02K80ZZ", "025T0ZZ", "025S0ZZ" |
| Codes used for Atrial Fibrillation ablation procedure | |
| Destruction of right atrium^@^ | 02563ZZ |
| Destruction of left atrium^@^ | 02573ZZ |
| Destruction of conduction mechanism^@^ | 02583ZZ |
| Destruction of Left Pulmonary Vein, Percutaneous Approach^@^ | 025T3ZZ |
| Destruction of Right Pulmonary Vein, Percutaneous Approach^@^ | 025S3ZZ |
| *^@^*Based on ICD-10 code, <https://www.icd10data.com> | |

**Supplementary Table 2:** ICD 10 codes for co-morbidities

| **Co-morbidities** | **ICD-10 Codes in Secondary field** |
| --- | --- |
| Obese^b^ | Utilized Elixhauser mapping program to find all obesity cases |
| BMI < 20 | Z681 |
| Hypertension^b^ | Utilized Elixhauser mapping program to find all hypertension cases |
| Diabetes^b^ | Utilized Elixhauser mapping program to find all diabetes cases |
| Hypercholesterolemia^@^ | [E78](https://www.icd10data.com/ICD10CM/Codes/E00-E89/E70-E88/E78-/E78) |
| Heart Failure^@^ | I50, I09.81, I97.13, I11.0, I13.0, I13.2 |
| Valvular disease^b^ | Utilized Elixhauser mapping program to find all valvular disease cases |
| Mitral Valve Stenosis^@^ | I34.2, I05.0/2 |
| Coronary Artery Disease^@^ | "I25", "I25.2", "I25.2", "I25.6" |
| COPD^@^ | J41, J42, J43, J44 |
| CKD stage 3 or more^@^ | "N183", "N1830", "N1831", "N1832", "N184", "N185", "N186" |
| History of CABG^@^ | I25.7, I25.8, I25.9, Z98.61 [Z95.5](https://www.icd10data.com/ICD10CM/Codes/Z00-Z99/Z77-Z99/Z95-/Z95.5), [T82.2](https://www.icd10data.com/ICD10CM/Codes/S00-T88/T80-T88/T82-/T82.2), Z95.1 |
| Hyperthyroidism^@^ | E05 |
| Cancer^b^ | Utilized Elixhauser mapping program to find all Cancer cases |
| Cardiomyopathy^@^ | I42 |
| Prior Stroke/TIA^@^ | I69.3, Z86.73 |
| Peripheral Vascular Disease^b^ | Utilized Elixhauser mapping program to find all Peripheral Vascular Disease cases |
| Autoimmune Disease^b^ | Utilized Elixhauser mapping program to find all Autoimmune Disease cases |
| ***^b^*** *Elixhauser Comorbidity Software Refined for ICD-10-CM* 2021, Agency for Healthcare Research and Quality R, Rockville, MD. [*www.hcup-us.ahrq.gov/toolssoftware/comorbidityicd10/comorbidityicd10.jsp*](http://www.hcup-us.ahrq.gov/toolssoftware/comorbidityicd10/comorbidityicd10.jsp).  *^@^*Based on ICD-10 code, <https://www.icd10data.com>. | |

**Supplementary Table 3:** ICD 10 coding (in secondary diagnosis field) for CHA_2_DS_2_-VASc and CHADS_2_ Score

| **CHA_2_DS_2_-VASc Score variables** | **ICD-10 codes** |
| --- | --- |
| Heart failure^@^- 1 point | I50, I09.81, I97.13, I11.0, I13.0, I13.2 |
| Hypertension^b^ – 1 point | Utilized Elixhauser mapping program to find all hypertension cases |
| Age < 65 years – 0 point | Age variables is provided in NIS database |
| Age >=65, <=75 years – 1 point |  |
| Age > 75 years – 2 points |  |
| Diabetes^b^ – 1 point | Utilized Elixhauser mapping program to find all diabetes cases |
| Vascular disease (Coronary, Aortic or any Peripheral vascular disease) ^@^ -1 point | I20, I21, I22, I23, I24, I25, T82.21, Z95.1, Z98.61, Z95.5, E08.5, E09.5, E10.5, E11.5, E13.5, I73, T82, Z98.62, Z95.820, I70, I71, I69, Z86, |
| Sex- Male-0 point | Sex information was provided in NIS database |
| Sex- Female- 1 point |  |
| History of TIA/Stroke- 2 points | I69.3, Z86.73 |
| *^b^ Elixhauser Comorbidity Software Refined for ICD-10-CM* 2021, Agency for Healthcare Research and Quality R, Rockville, MD. [*www.hcup-us.ahrq.gov/toolssoftware/comorbidityicd10/comorbidityicd10.jsp*](http://www.hcup-us.ahrq.gov/toolssoftware/comorbidityicd10/comorbidityicd10.jsp).  *^@^*Based on ICD-10 code, <https://www.icd10data.com> | |

**Supplementary Table 4:** ICD 10 coding for Complications

| **Complication** | **ICD-10 Codes** |
| --- | --- |
| - Pericardial Effusion*^@^* | I31.4, I31.2, I31.3 |
| - - Pericardial Drain*^@^* | 0W9D |
| - Cardiac Arrest*^@^* | “I46”, "I97120", "I97121", "I9712", "I97710" |
| - Post Procedure HF*^@^* | "I97130", "I97131" |
| Vascular Complication | |
| - Pseudoaneurysm*^@^* | "I724" |
| - Arterial Puncture*^@^* | "I9751" |
| - Arterial Embolism*^@^* | I74, K550, N280, H340, H341 |
| - Hematoma*^@^* | "I97410", "I97610", "I97630" |
| - Deep Vein Thrombosis*^@^* | “I82.4” |
| - Pulmonary Embolism*^@^* | "I2699", "I2609" |
| - AV Fistula*^@^* | "I770" |
| - Air Embolism*^@^* | "T790XXD", "T790XXS", "T800", "T800XXA", "T800XXD", "T800XXS" |
| Respiratory Complication | |
| - Diaphragmatic Paralysis*^@^* | "J986" |
| - Phrenic Nerve Injury*^@^* | "G588" |
| - Pneumothorax*^@^* | "J95811" |
| - Hemothorax*^@^* | “J942” |
| Circulatory Complication | |
| - Total Post Procedural Shock*^@^* | “T81” |
| - Major Bleeding *^@^* | “I61”, “I62”, “I69”, “K92”, “I92”, “I85”, “K22”, “K25”, “K29”, “K26”, “K27”, “K57”, “K51”, “K50”, “K62” |
| - - Bleeding Requiring Transfusion*^@^* | Major Bleeding + "30233H", "30233H0", "30233H1", 30233N", "30233N0","30233N1", "30233P", 30233P0", "30233P1", "30243N", “30243N0", "30243P", "30243N1", "30243P0", "30243P1", "30243H", "30243H0", “30243H1" |
| Neurological Complication | |
| - TIA*^@^* | “G45” |
| - Post Procedure Stroke*^@^* | "I97820", "I97810", "I9782" |
| Post-procedural sepsis*^@^* | “T81”, “T80”, “A40”, “A41”, “R65” |
| Anesthesia Complication*^@^* | T88 |
| *^@^*Based on ICD-10 code, <https://www.icd10data.com> | |
